# The association between tobacco use and COVID-19 in Qatar

**DOI:** 10.1016/j.pmedr.2022.101832

**Published:** 2022-05-19

**Authors:** Ahmad AlMulla, Ravinder Mamtani, Sohaila Cheema, Patrick Maisonneuve, Joanne Daghfal, Silva Kouyoumjian

**Affiliations:** a Tobacco Control Center, WHO Collaborative Center, Department of Medicine, Hamad Medical Corporation, P.O. Box 3050, Doha, Qatar; b Institute for Population Health, Weill Cornell Medicine-Qatar, P.O. Box 24144, Doha, Qatar; c Unit of Clinical Epidemiology, Division of Epidemiology and Biostatistics, IEO Istituto Europeo di Oncologia IRCSS, Milan, Italy; d Communicable Disease Center, Hamad Medical Corporation, P.O. Box 3050, Doha, Qatar

**Keywords:** COVID-19, Tobacco, Smoking, Smokeless, Electronic cigarettes, Qatar

## Abstract

The effects of smoking on COVID-19 are controversial. Some studies show no link between smoking and severe COVID-19, whereas others demonstrate a significant link. This cross-sectional study aims to determine the prevalence of tobacco use among COVID-19 patients, examine the relationship between tobacco use and hospitalized COVID-19 (non-severe and severe), and quantify its risk factors. A random sample of 7430 COVID-19 patients diagnosed between 27 February-30 May 2020 in Qatar were recruited over the telephone to complete an interviewer-administered questionnaire. The prevalence of tobacco smoking in the total sample was 11.0%, with 12.6% among those quarantined, 5.7% among hospitalized patients, and 2.5% among patients with severe COVID-19. Smokeless tobacco and e-cigarette use were reported by 3.2% and 0.6% of the total sample, respectively. We found a significant lower risk for hospitalization and severity of COVID-19 among current tobacco smokers (p < 0.001) relative to non-smokers (never and ex-smokers). Risk factors significantly related to an increased risk of being hospitalized with COVID-19 were older age (aged 55 + ), being male, non-Qatari, and those with heart disease, hypertension, diabetes, asthma, cancer, and chronic renal disease. Smokeless tobacco use, older age (aged 55 + ), being male, non-Qatari, previously diagnosed with heart disease and diabetes were significant risk factors for severe COVID-19. Our data suggests that only smokeless tobacco users may be at an increased risk for severe disease, yet this requires further investigation as other studies have reported smoking to be associated with an increased risk of greater disease severity.

## Highlights

• Tobacco smoking prevalence in the total sample was only 11.0%
• Smokeless tobacco users may be at an increased risk for severe disease.
• Smoking was not associated with an increased risk of greater disease severity.
• Increased age and co-morbidities were the most important risk factors for severity.
• Considering limitations, COVID-19 severity may be affected by other factors.

## 1 Introduction

The Coronavirus disease 2019 (COVID-19), caused by the Severe Acute Respiratory Syndrome Coronavirus 2 (SARS-CoV-2), was officially characterized as a pandemic by the World Health Organization (WHO) on 11 March 2020 (WHO, 2020). COVID-19 is predominately a disease of the respiratory tract characterized by severe acute respiratory syndrome. The virus uses angiotensin-converting enzyme 2 (ACE2) as a host cellular entry receptor, which is found in mucosal epithelial cells and the lung alveolar tissue (Zhou et al., 2020).

There is an ongoing debate about the link between tobacco use and the risk of contracting COVID-19. The act of smoking involves bringing the fingers to the mouth, which may increase the possibility of virus transmission if cigarettes, electronic devices, waterpipes, or fingers are contaminated with the virus (Simons and Brown, 2020). Long-term smoking may be a risk factor for COVID-19 due to the elevated expression of ACE2 among cigarette smokers (Li et al., 2020, Brake et al., 2020). Tobacco smoke exposure initiates inflammation in the lung, increases mucosal inflammation, causes mucus overproduction, and impaired mucociliary clearance (Strzelak et al., 2018). These processes may increase the risk of developing severe COVID-19 (Gupta et al., 2021).

The relationship between smoking and COVID-19 severity remains controversial with various studies publishing conflicting reports. Some studies show an increased risk of severe COVID-19 progression in smokers compared to non-smokers (Guan et al., 2019, Liu et al., 2020, Reddy et al., 2021). Smoking was associated with a poor prognosis of COVID-19 and serious adverse health outcomes, including death (Vardavas and Nikitara, 2020, Zhou et al., 2020, World Health Organization (WHO). WHO Statement: Tobacco Use and COVID-19. Available online at: https://www.who.int/news/item/11-05-, 2020). According to a recent *meta*-analysis, smokers were 1.91 times more likely to develop severe COVID-19 than non-smokers (Patanavanich and Glantz, 2020). Current research on the relationship between smoking and COVID-19 has yielded conflicting findings and some scholars have suggested that nicotine in tobacco may be considered as a potential treatment option for COVID-19 (Farsalinos et al., 2020, Farsalinos, K., Barbouni, A., Niaura, R. Systematic review of the prevalence of current smoking among hospitalized COVID-19 patients in China: could nicotine be a therapeutic option Reply. Intern Emerg Med., 2021, Polosa and Caci, 2020, Li Volti et al., 2020). However, the confusion is mainly driven by a lack of clarity about study endpoints. The literature available thus far is affected by several design issues, making study comparisons difficult. For instance, a positive association between smoking and risk of COVID-19-related severe outcomes including hospitalization and death was found in some studies (Hou et al., 2021, Clift et al., 2022, Williamson et al., 2020). Whereas, other studies have identified lower proportions of active smokers among patients diagnosed with COVID-19 (Della Valle et al., 2021, Hippisley-Cox et al., 2020, Rossato et al., 2020), and a significant lower prevalence of smoking among hospitalized COVID-19 patients than that expected on the basis of population smoking prevalence (Farsalinos et al., 2020, Farsalinos et al., 2021, Miyara et al., 2020).

In this study, we examine the possible relationship between COVID-19 and tobacco (smoking and smokeless) and/or electronic cigarette use during the ongoing pandemic. The Tobacco Control Center (TCC) WHO-Collaborating Center (WHO-CC) at Hamad Medical Corporation (HMC) aimed to test the hypothesis that tobacco and/or electronic cigarette users are at an increased risk of hospitalization and progression to severe COVID-19 in Qatar. We defined severe COVID-19 as those hospitalized patients who were admitted to the intensive care unit (ICU), received oxygenation, and needed mechanical ventilation or if the outcome was death. The other hospitalized patients were categorized as non-severe COVID-19 patients. Our objectives were the following: 1) determine the percentages of different types of tobacco and/or electronic cigarette use among all COVID-19 patients, 2) examine the relationship between tobacco (smoking and smokeless) and/or electronic cigarette use and hospitalization of COVID-19 (non-severe and severe), and 3) and quantify the risk factors for hospitalization and severe disease progression in COVID-19 patients.

## 2 Materials and methods

### 2.1 Study design and population

A telephone-based, cross-sectional study was conducted among patients diagnosed with COVID-19 who had been quarantined in selected hotels/accommodation facilities and among inpatients admitted to one of the hospitals of HMC for treatment ranging from general care to ICU. HMC is a large Joint Commission International-accredited group of nine tertiary hospitals across different regions in Qatar affiliated with the government and is exclusively responsible for all COVID-19 testing during the pandemic. Patients (aged 18 years and above) with a confirmed positive test result for COVID-19 nucleic acids by real-time fluorescence reverse transcription-polymerase chain reaction (RT-PCR) based on HMC laboratory testing were eligible to participate.

### 2.2 Sample size

The sample size was calculated using the most conservative hypothesized proportion of tobacco use (p = 0.50) with z = 2.58 for a 99% confidence interval and a margin of error of 1.40%. To account for a non-response of 20%, the minimum sample size required to estimate a population parameter was estimated at 10,158.

### 2.3 Recruitment

We randomly sampled all patients – regardless of their nationality – from the patient database of all COVID-19-positive cases diagnosed between February 27, 2020 and May 30, 2020 as provided by the Communicable Disease Center (CDC), HMC, Qatar (S1 Table in Supplementary Information [SI]). We aimed to represent all nationalities to achieve a balanced sample reflecting the population in Qatar as much as possible (Snoj, 2019). The contact information of COVID-19 patients was also provided by the CDC. Selected anonymous participants were recruited voluntarily to participate over the phone by telephone interviewers from the TCC. The telephone interviewers were blinded of any personal information of COVID-19 patients. At the time of the telephone survey these participants were either: (1) quarantined and recovered, (2) hospitalized and discharged, or (3) still in hospital and were reachable by phone. For deceased cases, tobacco and/or electronic cigarette-related information was extracted from the patient’s electronic health record. In some cases, we reached family members or friends/colleagues who shared accommodation with the deceased to answer the survey questions on their behalf.

### 2.4 Instrument and measures

A short telephone-based, questionnaire was developed with the support of the WHO Regional Office for the Eastern Mediterranean (WHO-EMRO). The questionnaire was piloted among members of the TCC HMC and deemed suitable for the study. The survey tool was available in Arabic and English (S1 File and S2 File of SI). Verbal consent was taken from everyone before completing the questionnaire. Agreeing to complete the telephone-based questionnaire was considered as informed consent by the participant to be included in the study. It took approximately five minutes to complete the questionnaire.

Demographic, tobacco, and clinical data of patients included in the study were collected over the telephone. Demographic data included gender, age, education, and premarital status. History and questions on current tobacco (smoking and smokeless) status and/or e-cigarette use were also asked. Questions related to second-hand exposure to tobacco smoking and vaping in the house and/or at the workplace and comorbidities (e.g. COPD, cancer, hypertension, diabetes) were also asked. In case of hospitalization, data about admission to the ICU, respiratory support (use of mechanical ventilation and oxygenation), and disease outcomes (recovery, discharge from hospital, or death) were also collected.

### 2.5 Data analysis

Collected data were coded and entered into Statistical Package for Social Sciences (IBM SPSS statistics; version 27; Armonk, NY: IBM Corporation program). Fifty percent of data entry was reviewed and repeated by a different individual to verify and validate the accuracy of the process. The first outcome variable was hospitalization of COVID-19 (non-severe and severe) and the second outcome variable was the severity of COVID-19 both expressed as a proportion (%). We defined severe COVID-19 as those hospitalized patients who were admitted to the ICU, received oxygenation, needed mechanical ventilation, or if the outcome was death. The other hospitalized patients were categorized as having non-severe COVID-19. The status of tobacco smoking by the respondents was divided into three categories: current smokers, ex-smokers, and never-smokers. For smokeless tobacco/e-cigarette use: current users, ex-users, and never users. Descriptive categorical variables were expressed as proportions. Statistical analysis for the association of variables with COVID-19 hospitalization and severity was carried out using the Chi-square test using a significance level of less than 5%. Variables that showed significant association at the bivariate level were entered into a multivariate logistic regression to identify the risk factors for COVID-19 hospitalization (non-severe and severe) and COVID-19 severe disease progression. Age, gender, nationality, tobacco smoking, smokeless tobacco use, heart disease, hypertension, diabetes, asthma, cancer, obesity, chronic renal disease, and other diseases were the factors entered into the first model. For the second model all the aforementioned factors were entered except asthma, cancer, and obesity.

### 2.6 Ethical consideration

The study procedures were approved by the Institutional Review Board at the Medical Research Center, HMC, Qatar.

## 3 Results

### 3.1 Sample characteristics

Out of the 10,158 selected study sample population, 151 were non-responsive and 2577 were unreachable. The final sample size included in the analysis was 7430 (S2 Table of SI), representing an overall survey response rate of 73.14%.

Socio-demographic, tobacco, and clinical characteristics of COVID-19 patients are shown in Table 1. The mean age of the sample was 38.8 ± 12.6 years; 71.0% (n = 5276) of them were below the age of 45 years and only 2.0% (n = 147) were over the age of 70 years. Of the sample, 73.5% (n = 5460) were men, 20.7% (n = 1539) were of Qatari nationality, 73.9% (n = 5440) were married, and 43.1% (n = 3158) had completed university-level education.

**Table 1:** Socio-demographic, tobacco, and clinical characteristics of COVID-19 patients.

	**All Patients^**	**Quarantined**	**Hospitalized**	**Non-severe COVID-19**	**Severe COVID-19**
**All subjects**	7430 (100.0)	5668 (100.0)	1762 (100.0)	1109 (100.0)	653 (100.0)
**Age (mean, ± SD)**	38.8 (±12.6)	36.3 (±11.0)	46.6 (±14.2)	44.4 (±13.9)	50.4 (±13.8)
**Age (years)**					
18–24	785 (10.6)	711 (12.5)	74 (4.2)	67 (6.0)	7 (1.1)
25–34	2419 (32.6)	2114 (37.3)	305 (17.3)	221 (19.9)	84 (12.9)
35–44	2072 (27.9)	1621 (28.6)	451 (25.6)	306 (27.6)	145 (22.2)
45–54	1248 (16.8)	818 (14.4)	430 (24.4)	258 (23.3)	172 (26.3)
55+	906 (12.2)	404 (7.1)	502 (28.5)	257 (23.2)	245 (37.5)
**Gender**					
Male	5460 (73.5)	4123 (72.7)	1337 (75.9)	779 (70.2)	558 (85.5)
Female	1970 (26.5)	1545 (27.3)	425 (24.1)	330 (29.8)	95 (14.5)
**Nationality**					
Qatari	1539 (20.7)	1210 (21.3)	329 (18.7)	262 (23.6)	67 (10.3)
Non-Qatari	5891 (79.3)	4458 (78.7)	1433 (81.3)	847 (76.4)	586 (89.7)
**Marital status**					
Single	1765 (24.0)	1536 (27.1)	229 (13.5)	191 (17.3)	38 (6.5)
Married	5440 (73.9)	4034 (71.2)	1406 (83.0)	877 (79.2)	529 (90.3)
Divorced/widowed	154 (2.1)	96 (1.7)	58 (3.4)	39 (3.5)	19 (3.2)
**Education**					
Secondary education	3830 (52.3)	2859 (50.5)	971 (58.6)	585 (52.8)	386 (70.2)
University	3158 (43.1)	2558 (45.2)	600 (36.2)	464 (41.9)	136 (24.7)
Postgraduate	332 (4.5)	245 (4.3)	87 (5.2)	59 (5.3)	28 (5.1)
**Tobacco Smoking**					
Yes	812 (11.0)	713 (12.6)	99 (5.7)	83 (7.5)	16 (2.5)
Ex-smoker	1423 (19.2)	995 (17.6)	428 (24.6)	273 (24.6)	155 (24.6)
Never-smoker	5171 (69.8)	3960 (69.9)	1211 (69.7)	753 (67.9)	458 (72.8)
**Smokeless use**					
Yes	235 (3.2)	179 (3.2)	56 (3.4)	27 (2.4)	29 (5.3)
Ex-user	292 (4.0)	203 (3.6)	89 (5.4)	44 (4.0)	45 (8.2)
Never-user	6796 (92.8)	5284 (93.3)	1512 (91.2)	1035 (93.6)	477 (86.6)
**E-cigarette user**					
Yes	42 (0.6)	34 (0.6)	8 (0.5)	8 (0.7)	0 (0.0)
Ex e-cigarette user	182 (2.5)	149 (2.6)	33 (2.0)	28 (2.5)	5 (0.9)
Never user	7081 (96.9)	5470 (96.8)	1611 (97.5)	1070 (96.7)	541 (99.1)
**Secondhand exposure**					
Smoke/smokeless tobacco used inside home	1040 (14.2)	796 (14.1)	244 (14.8)	170 (15.4)	74 (13.5)
No	6275 (85.8)	4865 (85.9)	1410 (85.2)	935 (84.6)	475 (86.5)
E-cigarettes used inside home	58 (0.8)	40 (0.7)	18 (1.1)	14 (1.3)	4 (0.7)
No	7257 (99.2)	5623 (99.3)	1636 (98.9)	1091 (98.7)	545 (99.3)
Smoking/vaping at workplace	543 (7.4)	410 (7.2)	133 (8.0)	79 (7.1)	54 (9.9)
No	5718 (78.2)	4497 (79.5)	1221 (73.8)	819 (74.1)	402 (73.4)
I do not work	1052 (14.4)	752 (13.3)	300 (18.1)	208 (18.8)	92 (16.8)
**Heart Diseases**					
Yes	162 (2.2)	52 (0.9)	110 (6.3)	38 (3.4)	72 (11.1)
No	7264 (97.8)	5615 (99.1)	1649 (93.7)	1071 (96.6)	578 (88.9)
**Hypertension**					
Yes	841 (11.3)	386 (6.8)	455 (25.9)	232 (20.9)	223 (34.3)
No	6585 (88.7)	5281 (93.2)	1304 (74.1)	877 (79.1)	427 (65.7)
**Diabetes**					
Yes	923 (12.4)	393 (6.9)	530 (30.1)	259 (23.4)	271 (41.7)
No	6503 (87.6)	5274 (93.1)	1229 (69.9)	850 (76.6)	379 (58.3)
**Asthma**					
Yes	244 (3.3)	163 (2.9)	81 (4.6)	55 (5.0)	26 (4.0)
No	7182 (96.7)	5504 (97.1)	1678 (95.4)	1054 (95.0)	624 (96.0)
**Chronic obstructive pulmonary disease**					
Yes	5 (0.1)	2 (0.0)	3 (0.2)	0 (0.0)	3 (0.5)
No	7421 (99.9)	5665 (100.0)	1756 (99.8)	1109 (100.0)	647 (99.5)
**Cerebrovascular disease**					
Yes	8 (0.1)	4 (0.1)	4 (0.2)	3 (0.3)	1 (0.2)
No	7418 (99.9)	5663 (99.9)	1755 (99.8)	1106 (99.7)	649 (99.8)
**Cancer**					
Yes	16 (0.2)	4 (0.1)	12 (0.7)	7 (0.6)	5 (0.8)
No	7410 (99.8)	5663 (99.9)	1747 (99.3)	1102 (99.4)	645 (99.2)
**Obesity**					
Yes	82 (1.1)	47 (0.8)	35 (2.0)	22 (2.0)	13 (2.0)
No	7344 (98.9)	5620 (99.2)	1724 (98.0)	1087 (98.0)	637 (98.0)
**Chronic renal disease**					
Yes	86 (1.2)	13 (0.2)	73 (4.2)	15 (1.4)	58 (8.9)
No	7340 (98.8)	5654 (99.8)	1686 (95.8)	1094 (98.6)	592 (91.1)
**Others**					
Yes	586 (7.9)	312 (5.5)	274 (15.6)	115 (10.4)	159 (24.5)
No	6840 (92.1)	5335 (94.5)	1485 (84.4)	994 (89.6)	491 (75.5)

^Total does not add up due to missing data. SD: Standard deviation.

### 3.2 Tobacco use and second-hand exposure

In the study sample, 11.0% (n = 812/7430) reported smoking tobacco (cigarettes, waterpipes, *medwakh* [Arabic traditional pipe]*,* and cigars); 19.2% (n = 1423) were ex-smokers; and 69.8% (n = 5171) were never-smokers (Table 1). The rate of tobacco smoking was 12.6% among the quarantined, 5.7% among hospitalized (non-severe and severe), and 2.5% in patients with severe COVID-19 (Table 1).

S3 Table of SI describes the number and percentage of different types of tobacco among smokers. Accordingly, of the current tobacco smokers (n = 812), 70.0% were cigarette smokers, 21.6% waterpipe, 2.6% *medwakh,* 0.4% cigar, 0.2% others, and 5.3% were smoking more than one type of tobacco.

For smokeless tobacco (*sweika/paan*), 3.2% (n = 235) reported current use, 4.0% (n = 292) were ex-users, and 92.8% (n = 6796) were never-users. The rate of smokeless tobacco was 3.2% among the quarantined, 3.4% among hospitalized, and 5.3% among patients with severe COVID-19. For e-cigarettes, 0.6% (n = 42) reported using e-cigarette, 2.5% (n = 182) were ex-users and 96.9% (n = 7081) were never-users.

Compared to non-smokers and ex-smokers, current smokers were predominantly male, younger, and reported less chronic diseases. For instance, 12.2% of smokers were hospitalized vs. 23.4% of non-smokers (S4 Table of SI). Moreover, a lower number of smokers were admitted to ICU comparing to non-smokers (1.8% vs. 8.6%), received oxygenation (3.2% vs. 11.5%) and mechanical ventilation (1.1% vs. 4.3%), and a smaller number of deaths were reported among smokers (0.9% vs. 1.5%).

Smokeless users were similar in age to non-smokeless users and ex-users (S5 Table of SI). Compared to non-users, smokeless users were more likely to be hospitalized (23.8% vs. 22.2%), admitted to the ICU (11.5% vs. 6.9%), receive oxygenation (13.2% vs. 9.6%) and mechanical ventilation (8.5% vs. 3.1%). Additional details of sociodemographic and clinical characteristics of COVID-19 patients by tobacco smoking, smokeless tobacco, and electronic cigarette use are shown in S4-S6 Tables of SI.

Second-hand exposure of tobacco smoking inside the house was reported among 14.2% of the total sample, with 0.8% (n = 58) reporting e-cigarette use inside their homes. Smoking or vaping in indoor areas at the workplace was reported among 8.7% (n = 543), with 14.4% (n = 1052) reporting that they do not work, and 78.2% (n = 5718) stating they were not exposed in work.

### 3.3 Pre-existing conditions

Regarding pre-existing conditions, of the total sample study 2.2% (n = 162) reported having heart disease, 11.3% (n = 841) hypertension, 12.4% (n = 923) diabetes, 3.3% (n = 244) asthma, 1.2% (n = 86) chronic renal disease, 0.1% (n = 5) COPD, 0.1% (n = 8) cardiovascular disease (CVD), 0.22% (n = 16) cancer, 1.1% (n = 82) obesity, and others 7.9% (n = 586). Additional details are provided in S7 Table of SI.

### 3.4 Hospitalized and severe COVID-19 and their related factors

Out of the total sample, 76.3% of individuals were quarantined and recovered without hospitalization (n = 5668), while 23.7% (n = 1762) were hospitalized (Table 1). Out of the n = 1762 individuals hospitalized, 92.8% were discharged after recovery (n = 1636), 0.4% were still hospitalized at the time of their interview (n = 7), and 6.8% had died (n = 119). Of the hospitalized patients (non-severe and severe; n = 1762), 36% were admitted to the ICU (n = 635), 47.6% received oxygen support (n = 839), 18.6% received mechanical ventilation (n = 328), and 6.8% were reported as death cases (n = 119). Out of the total hospitalized patients, 62.9% individuals had non-severe COVID-19 (n = 1109), while 37.1% had severe COVID-19 (n = 653). Sample characteristics of COVID-19 patients quarantined versus hospitalized and by COVID-19 severity (non-severe versus severe) are provided in Table 1.

In bivariate analyses, only the variables that were significantly associated with hospitalization of COVID-19 (non-severe and severe) and severe disease progression of COVID-19 were included in both models, respectively (Table 1). The first model was found to be statistically significant, χ2 (18, N = 7430) = 1042.2, p < 0.001, −2LL = 6788.99, and 0.2 Nagelkerke pseudo-R square. In multivariate analysis (Table 2), the odds of hospitalization of COVID-19 patients were higher among older age groups, particularly five times higher among those aged 55 years old and above (adjusted odds ratio (AOR) = 4.9, 95% Confidence interval (CI) 3.6–6.5), males (AOR = 1.2, 95% CI 1.1–1.4), non-Qataris (AOR = 1.3, 95% CI 1.1–1.5), and among those with heart diseases (AOR = 2.0, 95% CI 1.4–3.0), hypertension (AOR = 1.7, 95% CI 1.4–2.0), diabetes (AOR = 2.5, 95% CI 2.1–3.0), asthma (AOR = 1.6, 95% CI 1.2–2.2), cancer (AOR = 5.0, 95% CI 1.5–17.2), and chronic renal diseases (AOR = 4.2, 95% CI 2.0–8.9). Tobacco smokers had lower odds of being hospitalized for COVID-19 (AOR = 0.5, 95% CI 0.4–0.7) than non-smokers. Compared to non-users, the odds of hospitalization were higher only among ex-users of smokeless tobacco (AOR = 1.5, 95% CI 1.1–2.0).

**Table 2:** Multivariate logistic regression of hospitalized and severe patients.

**Characteristic**	**Coef**	**SE Coef**	**Hospitalized COVID-19** **Adjusted Odds Ratio (AOR) (95% CI)**	p-value*	**Coef**	**SE Coef**	**Severe Covid-19** **Adjusted Odds Ratio (AOR) (95% CI)**	p-value*
Age								
18–24	–	–	1.00 (Ref.)	–	–	–	1.00 (Ref.)	–
25–34	0.2	0.1	1.3 (0.9–1.7)	0.082	1.2	0.5	3.3 (1.3–8.2)	0.010
35–44	0.7	0.1	2.1 (1.6–2.7)	<0.001	1.1	0.5	3.0 (1.2–7.4)	0.015
45–54	1.1	0.1	3.0 (2.2–3.9)	<0.001	1.2	0.5	3.3 (1.3–8.1)	0.009
55+	1.6	0.2	4.9 (3.6–6.5)	<0.001	1.4	0.5	3.9 (1.6–9.7)	0.003
Gender								
Female	–	–	1.00 (Ref.)	–	–	–	1.00 (Ref.)	–
Male	0.2	0.1	1.2 (1.1–1.4)	0.022	0.9	0.2	2.6 (1.9–3.5)	<0.001
Nationality								
Qatari	–	–	1.00 (Ref.)	–	–	–	1.00 (Ref.)	–
Non-Qatari	0.2	0.1	1.3 (1.1–1.5)	0.005	0.9	0.2	2.6 (1.9–3.7)	<0.001
Tobacco Smoking								
Never-smoker	–	–	1.00 (Ref.)	–	–	–	1.00 (Ref.)	–
Smoker	−0.7	0.1	0.5 (0.4–0.7)	<0.001	−1.6	0.4	0.2 (0.1–0.5)	<0.001
Ex-smoker	0.1	0.1	1.1 (1.0–1.3)	0.10	−0.4	0.1	0.7 (0.5–0.9)	0.008
Smokeless (sweika/paan)								
Never-user	–	–	1.00 (Ref.)	–	–	–	1.00 (Ref.)	–
User	0.1	0.2	1.1 (0.8–1.6)	0.55	0.8	0.3	2.0 (1.1–3.7)	0.019
Ex-user	0.4	0.1	1.5 (1.1–2.0)	0.009	0.6	0.2	1.8 (1.1–2.9)	0.014
Heart Diseases								
No	–	–	1.00 (Ref.)	–	–	–	1.00 (Ref.)	–
Yes	0.7	0.2	2.0 (1.4–3.0)	<0.001	0.7	0.3	2.0 (1.2–3.3)	0.007
Hypertension								
No	–	–	1.00 (Ref.)	–	–	–	1.00 (Ref.)	–
Yes	0.5	0.1	1.7 (1.4–2.0)	<0.001	0.2	0.1	1.2 (0.9–1.6)	0.15
Diabetes								
No	–	–	1.00 (Ref.)	–			1.00 (Ref.)	–
Yes	0.9	0.1	2.5 (2.1–3.0)	<0.001	0.6	0.1	1.7 (1.3–2.3)	<0.001
Asthma								
No	–	–	1.00 (Ref.)	–	–	–	–	–
Yes	0.5	0.2	1.6 (1.2–2.2)	0.002	–	–	–	–
Cancer								
No			1.00 (Ref.)	–	–	–	–	–
Yes	1.6	0.6	5.0 (1.5–17.2)	0.010	–	–	–	–
Obesity								
No	–	–	1.00 (Ref.)	–	–	–	–	–
Yes	0.4	0.3	1.5 (0.9–2.5)	0.14	–	–	–	–
Chronic renal disease								
No	–	–	1.00 (Ref.)	–	–	–	1.00 (Ref.)	–
Yes	1.4	0.4	4.2 (2.0–8.9)	<0.001	0.6	0.4	1.8 (0.8–4.0)	0.16
Other diseases								
No	–	–	1.00 (Ref.)	–	–	–	1.00 (Ref.)	–
Yes	0.6	0.1	1.9 (1.5–2.3)	<0.001	0.7	0.2	2.1 (1.5–2.9)	<0.001

* Factors with p-value < 0.05 were considered statistically significant. Abbreviations: Coef: Coefficient; SE: Standard error.

The second model was found to be statistically significant, χ2 (15, N = 1762) = 234.4, p < 0.001, −2LL = 1873.1, and 0.2 Nagelkerke pseudo-R square. In multivariate analysis (Table 2), odds of severe COVID-19 were higher among older age groups, particularly four times higher among those aged 55 years old and above (AOR = 3.9, 95% CI 1.6–9.7), males (AOR = 2.6, 95% CI 1.9–3.5), non-Qataris (AOR = 2.6, 95% CI 1.9–3.7), and among those with heart diseases (AOR = 2.0, 95% CI 1.2–3.3), and diabetes (AOR = 1.7, 95% CI 1.3–2.3). Odds of severe COVID-19 were significantly lower among tobacco smokers (AOR = 0.2, 95% CI 0.1–0.5) than non-smokers. However, for smokeless users and ex-users, the odds of severe COVID-19 were significantly higher (AOR = 2.0, 95% CI 1.1–3.7 and AOR = 1.8, 95% CI 1.1–2.9) than non-users.

Among the hospitalized smokeless tobacco users, 51.8% (n = 29) were severe COVID-19 patients. The common nationalities were Asian countries as mentioned in S8 Table of SI. The outcome among severe cases of hospitalized smokeless tobacco users showed that 86.2% (n = 25) were discharged from the hospital after recovery, 3.4% (n = 1) remained hospitalized, and 10.3% were deceased (n = 3) (S9 Table of SI). However, there were no significant differences in the mean number of days spent in the hospital (p = 0.83), the ICU (p = 0.81), and those who received mechanical ventilation (p = 0.62) among smokeless tobacco users, ex-users, and non-users with severe COVID-19 (S10 Table of SI).

We investigated the effects of smoking in young patients aged 18–49 years and over 50 years. These multivariable stratified analyses presented reduced risk of hospitalization associated with tobacco smoking in both age groups, but the inverse association of smokeless tobacco with severe disease was present for patients aged < 50 years only. More details can be found in S11 Table and S12 Table of SI, respectively.

## 4 Discussion

To our knowledge, this is the first study in Qatar and in the WHO’s Eastern Mediterranean Region (EMR) examining the relationship between tobacco (smoking and smokeless) and/or electronic cigarettes and COVID-19. Our study demonstrates that the rate of smoking among COVID-19 patients is lower (11.0%) than the overall rate of smoking in Qatar’s population. According to a recent study, the rate of overall tobacco use among adults in Qatar was 25.2%, with 21.5% being tobacco smokers (AlMulla et al., 2021). The smoking rate among COVID-19 patients reported in the literature is consistently lower than the population average (Alqahtani et al., 2020). Similarly, a recent *meta*-analysis reported a low prevalence of current smoking (5.7%) among hospitalized COVID-19 patients (Farsalinos et al., 2020). It might be that smokers adhere more to preventive measures, such as masking, social distancing, and/or other factors.

In our study, we found a significant lower risk for hospitalization and for severe COVID-19 among current tobacco smokers compared to non-smokers. However, smokeless tobacco use was associated with a greater risk for severe COVID-19 (AOR = 2.0, 95% CI1.1–3.7). This is in line with published literature, which shows that all forms of tobacco increase the risk of mortality and serious complications (Gupta et al., 2021, Alqahtani et al., 2020, Gülsen et al., 2020, Tobacco Control, 2021, Gaunkar et al., 2020). Several studies have described a significant nicotine-induced reduction of membrane ACE-2 protein expression and an anti-inflammatory response due to nicotinic acetylcholine receptor and suggested a therapeutic value in COVID-19 patients (Caruso et al., 2021, Leung et al., 2020, Russo et al., 2020, Farsalinos et al., 2020). However, according to a review, smokeless tobacco use seems to worsen the progression and prognosis of COVID-19 by nicotine-induced increased expression of the ACE2 receptor and action of the furin enzyme in the oral cavity (Gaunkar et al., 2020). The South Asian region accounts for the highest global consumption of smokeless tobacco. This was reflected in our study, in which most of the severe COVID-19 smokeless tobacco users were coming from Nepal (55.2%), Bangladesh (13.8%), India (10.3%), and Pakistan (13.8%) (S8 Table of SI). This can be explained, as Asian migrant workers comprise a vast majority of the expatriate labor population in Qatar. Moreover, during the early phase of the pandemic, most of the COVID-19-reported cases were linked to migrant workers who were quarantined and tested as a precautionary measure to combat virus spread, and who constituted almost one-fifth of our sample (Table S2 of SI).

Increased age (over 70 years) is considered the most important risk factor for COVID-19 severity and mortality (Docherty et al., 2020). In one of the largest cohort studies conducted in England, Scotland and Wales including 20,133 patients, the median age of patients admitted to hospital with COVID-19 or with a diagnosis of COVID-19 made in hospital was 73 years (Docherty et al., 2020). However, in our study, only two percent of our sample were aged over 70 years. This large difference may be responsible for the divergent result of the protective association of tobacco smoking with COVID-19 severity. This is a peculiarity of the population in Qatar, given the young age of the population, which has led to a relatively small number of deaths (119/7430 = 1.6%) compared to other countries (Docherty et al., 2020, Grasselli et al., 2020, Petrilli et al., 2019) in which a larger proportion of the population is aged over 70 years.

In our study, individuals were more vulnerable to hospitalization and severe COVID-19 with increasing age, as reported that the elderly and those with pre-existing multi-morbid conditions may be at higher risk of developing severe health consequences from COVID-19 (Matsushita et al., 2020). We found that primarily heart disease and diabetes were risk factors for severe COVID-19, similar to previously published literature (Gupta et al., 2021, Zhong et al., 2021, Guo et al., 2020, Huang et al., 2020). Smoking is a well-recognized risk factor for pre-existing comorbidities such as cardiovascular disease and diabetes, which seem to contribute to worse outcomes (Salah et al., 2020). It may be that smoking played some sort of mediational role in the development of severe disease. Of the total sample, the higher prevalence of co-morbidities (heart disease, hypertension, and diabetes) among smokeless tobacco users might explain why they were at an increased risk of severe COVID-19. Moreover, this subgroup was older in age compared to smokers. Yet, further investigation is warranted.

There is a close relationship between respiratory diseases, smoking and ACE2 modulation that could increase the risk for developing severe COVID-19 outcomes (Sanchez-Ramirez and Mackey, 2020). However, our study reported a very low prevalence of COPD (0.1%), much lower than the literature (Alqahtani et al., 2020, Singh et al., 2020). As previously discussed, this may be because our sample mainly consists of the younger population in Qatar, with a mean age of 38.8 (±12.6) years. COPD is considered a disease of the elderly and is not common in young adults and usually diagnosed after the age of 45 years (Holm et al., 2014).

In our study, men had higher risk of being hospitalized and higher risk of severe COVID-19, consistent with published studies (Guan et al., 2019, Zhong et al., 2021, Abate et al., 2020, Yang et al., 2020). Additionally, based on a global large-scale *meta*-analysis, a higher severity of COVID-19 in male gender was associated with an odds ratio of 2.84, like what was reported in our study (AOR = 2.6, 95% CI 1.9–3.5). Some studies proposed that higher risk among men can be due to smoking; in our study, the smoking rate was higher among males (13.8% vs. 2.8%) and similarly among smokeless tobacco and e-cigarette users. However, there might be other unidentified factors playing a role.

Similar to our findings, several other studies reported low odds of testing positive for COVID-19 and of disease severity among current smokers (Israel et al., 2020, Rentsch et al., 2020, Prinelli et al., 2021, Tomaselli et al., 2022, Simons et al., 2021) or found negative or no relationship between the severity of COVID-19 and smoking (Zhong et al., 2021, Tsigaris and Teixeira da Silva, 2020). However, tobacco smoking should not be considered as an efficient protection against COVID-19, as has been suggested (Lippi and Henry, 2020, Paleiron et al., 2021). There is insufficient evidence to confirm any link between tobacco or nicotine use for the prevention or treatment of COVID-19 (World Health Organization (WHO). WHO Statement: Tobacco Use and COVID-19. Available online at: https://www.who.int/news/item/11-05-, 2020, World Health Organization (WHO). Smoking and COVID-19. Scientific Brief. available at https://www.who.int/news-room/commentaries/detail/smoking-and-covid-19. Accessed on 7 Febraury, 2021). On the contrary, smokers appear to have an increased risk of hospitalization and severe COVID-19 (Simons et al., 2020, Lowe et al., 2021, Emami et al., 2020).

The COVID-19 pandemic presents a unique opportunity to motivate tobacco users to quit and to offer advice, medication, and support by offering evidence-based tobacco cessation services. Tobacco dependence treatment services across Qatar provide full cost cessation support and treatment for all Qataris and non-Qataris (AlMulla et al., 2019); and, in response to the pandemic, the Tobacco Control Center WHO Collaborating Center in Hamad Medical Corporation provided tobacco cessation services by phone consultations (AlMulla and Kouyoumjian, 2021). Smoking cessation services providing counseling and pharmacological interventions are proven to be cost-effective and improve success rates compared to unaided quit attempts (Aveyard et al., 2012, Zwar et al., 2014). Fortunately, a recent study among healthcare workers in Qatar showed that almost 60% of them provided tobacco cessation interventions to patients (AlMulla et al., 2021).

In this cross-sectional study, COVID-19 patients who smoked or used tobacco accounted for a relatively small proportion of the total sample, and the relationship between these behaviors and the severity of COVID-19 may be affected by other confounding factors. It is also possible that some patients may have been in critical condition at the time they were hospitalized, which could have compromised their communication capacity and their ability to report their correct smoking status, particularly for the deceased cases. The study sample does not reflect the true proportion of nationality stratification in the country; therefore, the results may not be generalizable to the wider population.

## 5 Conclusion

This study indicates that only smokeless tobacco users may be at an increased risk for severe disease; however, this requires further investigation as other studies have reported smoking to be associated with an increased risk of greater disease severity. Smoking prevention initiatives among never smokers should be promoted, and healthcare practitioners must continue to identify, advise, and engage smokers/users and support them in cessation efforts. Patients with underlying disease, such as diabetes and heart disease, should follow preventive measures for COVID-19, such as social distancing, facemask use, avoiding social gatherings, and vaccination and must be monitored for COVID-19 and disease progression.

## 6 Authors’ contributions

AA, the principal investigator, conceived and led the design of the study, provided input during all stages of the study, and reviewed the final draft of the manuscript. RM and SC collectively contributed to reviewing and drafting the article. PM contributed to data analyses and reviewing the article. SK performed data analyses, wrote the first draft of the manuscript, and contributed to the design of study questionnaire. JD retrieved data from the COVID-19 database. All authors contributed to discussion and interpretation of the results and the writing of the manuscript. All authors have read and approved the final manuscript.

## Declaration of Competing Interest

The authors declare that they have no known competing financial interests or personal relationships that could have appeared to influence the work reported in this paper.
