# Supplementary material for: The association between tobacco use and COVID-19 in Qatar
Source: Prev Med Rep. 2022 May 19;28:101832. doi: 10.1016/j.pmedr.2022.101832 (PMC9116971; doi:10.1016/j.pmedr.2022.101832)
Supplement: Supplementary Data 1 [file mmc1.docx]

Supplementary Information

**The association between tobacco use and COVID-19 in Qatar**

Ahmad AlMulla,^1^ Ravinder Mamtani,^2^ Sohaila Cheema,^2^ Patrick Maisonneuve,^3^ Joanne Daghfal,^4^ Silva Kouyoumjian^1*^

^1^ *Tobacco Control Center, WHO Collaborative Center, Department of Medicine, Hamad Medical Corporation, P.O. Box 3050, Doha, Qatar.*

*^2^ Institute for Population Health, Weill Cornell Medicine-Qatar, P.O. Box 24144, Doha, Qatar.*

^3^ *Unit of Clinical Epidemiology, Division of Epidemiology and Biostatistics, IEO Istituto Europeo di Oncologia IRCSS, Milan, Italy.*

*^4^ Communicable Disease Center, Hamad Medical Corporation, P.O. Box 3050, Doha, Qatar*

**S1 Table**. The proportion of the study sample conveniently selected and responded by nationality

| **Nationality** | **Sample Frame** | **N (number of individuals selected)** | **%** | **N (number of individuals responded)** | **%** |
| --- | --- | --- | --- | --- | --- |
| Qatari | 2997 | 1900 | 18.70 | 1539 | 20.71 |
| Philippines | 2877 | 999 | 9.83 | 723 | 9.73 |
| Sudanese | 1125 | 769 | 7.57 | 667 | 8.98 |
| Egyptian | 2778 | 786 | 7.74 | 601 | 8.09 |
| Indian | 18221 | 672 | 6.62 | 470 | 6.33 |
| Somali | 800 | 441 | 4.34 | 145 | 1.95 |
| Nepalese | 11366 | 552 | 5.43 | 232 | 3.12 |
| Bangladesh | 9140 | 564 | 5.55 | 287 | 3.86 |
| Iranian | 396 | 324 | 3.19 | 197 | 2.65 |
| Yemeni | 370 | 292 | 2.87 | 228 | 3.07 |
| Jordanian | 341 | 294 | 2.89 | 235 | 3.16 |
| Syrian | 325 | 272 | 2.68 | 175 | 2.36 |
| Palestinian | 310 | 264 | 2.60 | 186 | 2.50 |
| Kenyan | 747 | 245 | 2.41 | 130 | 1.75 |
| Pakistani | 3823 | 329 | 3.24 | 253 | 3.41 |
| Sri Lankan | 2072 | 209 | 2.06 | 186 | 2.50 |
| Others | 2616 | 1246 | 12.27 | 1176 | 15.83 |
| **Total** | **60,304** | **10,158** | **100.00** | **7,430** | **100.00** |

**S1 File.** The study questionnaire in English

**S2 File.** The study questionnaire in Arabic

**S2 Table.** Respondents by nationality – proportion of study sample

| **Nationality** | **n** | **%** |
| --- | --- | --- |
| Qatar | 1539 | 20.71 |
| India | 470 | 6.33 |
| Bangladesh | 287 | 3.86 |
| Nepal | 232 | 3.12 |
| Pakistan | 253 | 3.41 |
| Sri Lanka | 186 | 2.50 |
| Philippines | 723 | 9.73 |
| Egypt | 601 | 8.09 |
| Sudanese | 667 | 8.98 |
| Jordanian | 235 | 3.16 |
| Yemeni | 228 | 3.07 |
| Iranian | 197 | 2.65 |
| Palestinian | 186 | 2.50 |
| Syrian | 175 | 2.36 |
| Tunisian | 154 | 2.07 |
| Somali | 145 | 1.95 |
| Moroccan | 106 | 1.43 |
| Kenyan | 130 | 1.75 |
| Others* | 916 | 12.33 |
| **Total** | **7430** | **100.00** |

*Includes other 78 nationalities

**S3 Table.** The number and percentage of different types of current tobacco smoking by nationality (n, %) smoking refers to tobacco smoking (cigarettes, waterpipe, *medwakh* and cigar)

|  | **All tobacco users (n=812)** | | |
| --- | --- | --- | --- |
| Types of tobacco use | Any use  n (%) | Exclusive use  n (%) | Concomitant with other type  n (%) |
| **Tobacco smoking** |  |  |  |
| Cigarette | 609 (74.9) | 568 (70.0) | 41 (5.0) |
| Waterpipe | 209 (25.8) | 175 (21.6) | 34 (4.2) |
| *Medwakh* | 33 (4.1) | 21 (2.6) | 12 (1.5) |
| Cigar | 7 (0.6) | 3 (0.4) | 4 (0.5) |
| Others (pipe, IQOS^*^) | 3 (0.4%) | 2 (0.2) | 1 (0.1) |
| **More than one type** |  |  | 43 (5.3) |

*IQOS: I quit original smoking

**S4 Table.** Sociodemographic and clinical characteristics of COVID-19 patients by smoking status

|  | **COVID-19 patients** | | | |
| --- | --- | --- | --- | --- |
|  | All Patients^ | Smokers* | Non-smokers | Ex-smokers |
|  | (n, % of total) | (n, %) | (n, %) | (n, %) |
| All subjects | 7430 (100%) | 812 (11.0%) | 5171 (69.8%) | 1423 (19.2%) |
| Age (mean, ± SD) | 38.8 (±12.6) | 34.9 (±9.5) | 38.5 (±12.8) | 41.5 (±12.4) |
| Age (years) |  |  |  |  |
| 18-24 | 785 (10.6%) | 94 (11.6%) | 611 (11.8%) | 79 (5.6%) |
| 25-34 | 2419 (32.6%) | 369 (45.4%) | 1649 (31.9%) | 400 (28.1%) |
| 35-44 | 2072 (27.9%) | 220 (27.1%) | 1435 (27.8%) | 416 (29.2%) |
| 45-54 | 1248 (16.8%) | 99 (12.2%) | 844 (16.3%) | 297 (20.9%) |
| 55+ | 906 (12.2%) | 30 (3.7%) | 632 (12.2%) | 231 (16.2%) |
| Gender |  |  |  |  |
| Male | 5460 (73.5%) | 756 (93.1%) | 3358 (64.9%) | 1323 (93.0%) |
| Female | 1970 (26.5%) | 56 (6.9%) | 1813 (35.1%) | 100 (7.0%) |
| Nationality |  |  |  |  |
| Qatari | 1539 (20.7%) | 161 (19.8%) | 1156 (22.4%) | 221 (15.5%) |
| Non-Qatari | 5891 (79.3%) | 651 (80.2%) | 4015 (77.6%) | 1202 (84.5%) |
| Marital status |  |  |  |  |
| Single | 1765 (24.0) | 265 (32.8%) | 1228 (23.9%) | 271 (19.1%) |
| Married | 5440 (73.9%) | 534 (66.2%) | 3777 (73.6%) | 1128 (79.6%) |
| Divorced/widowed | 154 (2.1%) | 8 (1.0%) | 128 (2.5%) | 18 (1.3%) |
| Education |  |  |  |  |
| Secondary schooling or less | 3830 (52.3%) | 404 (50.2%) | 2649 (52.0%) | 777 (54.9%) |
| University | 3158 (43.1%) | 353 (43.9%) | 2234 (43.8%) | 571 (40.3%) |
| Postgraduate | 332 (4.5%) | 48 (6.0%) | 216 (4.2%) | 68 (4.8%) |
| Secondhand exposure |  |  |  |  |
| Smoke/smokeless inside home | 1040 (14.2%) | 201 (25.1%) | 615 (12.1%) | 224 (15.9%) |
| No | 6275 (85.8%) | 601 (74.9%) | 4486 (87.9%) | 1188 (84.1%) |
|  |  |  |  |  |
| E-cigarettes inside home | 58 (0.8%) | 10 (1.2%) | 32 (0.6%) | 16 (1.1%) |
| No | 7257 (99.2%) | 792 (98.8%) | 5070 (99.4%) | 1397 (98.9%) |
|  |  |  |  |  |
| Smoking/vaping at workplace | 543 (8.7%) | 85 (10.6%) | 305 (6.0%) | 153 (10.8%) |
| No | 5718 (78.2%) | 660 (82.2%) | 3924 (77.0%) | 1134 (80.3%) |
| I do not work | 1052 (14.4%) | 58 (7.2%) | 868 (17.0%) | 126 (8.9%) |
| Heart Diseases |  |  |  |  |
| Yes | 162 (2.2%) | 9 (1.1%) | 108 (2.1%) | 45 (3.2%) |
| No | 7264 (97.8%) | 803 (98.9%) | 5060 (97.9%) | 1378 (96.8%) |
| Hypertension |  |  |  |  |
| Yes | 841 (11.3%) | 41 (5.0%) | 569 (11.0%) | 227 (16.0%) |
| No | 6585 (88.7%) | 771 (95.0%) | 4599 (89.0%) | 1196 (84.0%) |
| Diabetes |  |  |  |  |
| Yes | 923 (12.4%) | 57 (7.0%) | 629 (12.2%) | 228 (16.0%) |
| No | 6503 (87.6%) | 755 (93.0%) | 4539 (87.8%) | 1195 (84.0%) |
| Asthma |  |  |  |  |
| Yes | 244 (3.3%) | 23 (2.8%) | 176 (3.4%) | 45 (3.2%) |
| No | 7182 (96.7%) | 789 (97.2%) | 4992 (96.6%) | 1378 (96.8%) |
| COPD |  |  |  |  |
| Yes | 5 (0.1%) | 1 (0.1%) | 3 (0.1%) | 1 (0.1%) |
| No | 7421 (9.9%) | 811 (99.9%) | 5165 (99.9%) | 1422 (99.9%) |
| Cerebrovascular disease |  |  |  |  |
| Yes | 8 (0.1%) | 0 (0.0%) | 3 (0.1%) | 5 (0.4%) |
| No | 7418 (99.9%) | 812 (100.0%) | 5165 (99.9%) | 1418 (99.6%) |
| Cancer |  |  |  |  |
| Yes | 16 (0.2%) | 1 (0.1%) | 10 (0.2%) | 5 (0.4%) |
| No | 7410 (99.8%) | 811 (99.9%) | 5158 (99.8%) | 1418 (99.6%) |
| Obesity |  |  |  |  |
| Yes | 82 (1.1%) | 7 (0.9%) | 62 (1.2%) | 13 (0.9%) |
| No | 7344 (98.9%) | 805 (99.1%) | 5106 (98.8%) | 1410 (99.1%) |
| Chronic renal disease |  |  |  |  |
| Yes | 86 (1.2%) | 4 (0.5%) | 58 (1.1%) | 13 (0.9%) |
| No | 7340 (98.8%) | 808 (99.5%) | 5110 (98.9%) | 1410 (99.1%) |
| Others |  |  |  |  |
| Yes | 586 (7.9%) | 50 (6.2%) | 405 (7.8%) | 119 (8.4%) |
| No | 6840 (92.1%) | 762 (93.8%) | 4763 (92.2%) | 1304 (91.6%) |
| Quarantine/Hospitalized |  |  |  |  |
| Quarantine | 5668 (76.3%) | 713 (87.8%) | 3960 (76.6%) | 995 (69.9%) |
| Hospitalized | 1762 (23.7%) | 99 (12.2%) | 1211 (23.4%) | 428 (30.1%) |
| Intensive Care Unit |  |  |  |  |
| Yes | 635 (8.5%) | 15 (1.8%) | 444 (8.6%) | 154 (10.8%) |
| No | 6795 (91.5%) | 797 (98.2%) | 4727 (91.4%) | 1269 (89.2%) |
| Oxygen therapy |  |  |  |  |
| Yes | 839 (11.3%) | 26 (3.2%) | 593 (11.5%) | 197 (13.8%) |
| No | 6591 (88.7%) | 786 (96.8%) | 4578 (88.5%) | 1226 (86.2%) |
| Mechanical ventilation |  |  |  |  |
| Yes | 328 (4.4%) | 9 (1.1%) | 222 (4.3%) | 79 (5.6%) |
| No | 7102 (95.6%) | 803 (98.9%) | 4949 (95.7%) | 1344 (94.4%) |
| Outcome |  |  |  |  |
| Recovered without hospitalization | 5668 (76.3%) | 713 (87.8%) | 3960 (76.6%) | 995 (69.9%) |
| Recovered with hospitalization | 1636 (22.0%) | 92 (11.3%) | 1126 (21.8%) | 418 (29.4%) |
| Still in hospital | 7 (0.1%) | 0 (0.0%) | 6 (0.1%) | 1 (0.1%) |
| Death | 119 (1.6%) | 7 (0.9%) | 79 (1.5%) | 9 (0.6%) |

SD: Standard deviation *Includes daily and occasional users. ^Total does not add up due to missing data.

**S5 Table.** Sociodemographic and clinical characteristics of COVID-19 patients by smokeless tobacco use status

|  | **COVID-19 patients** | | | |
| --- | --- | --- | --- | --- |
|  | All Patients^ | Smokeless users* | Smokeless non-users | Smokeless ex-users |
|  | (n, % of total) | (n, %) | (n, %) | (n, %) |
| All subjects | 7430 (100%) | 235 (3.2%) | 6796 (92.8%) | 292 (4.0%) |
| Age (mean, ± SD) | 38.8 (±12.6) | 38.2 (±11.1) | 38.5 (±12.4) | 38.0 (±11.1) |
| Age (years) |  |  |  |  |
| 18-24 | 785 (10.6%) | 18 (7.7%) | 741 (10.9%) | 24 (8.2%) |
| 25-34 | 2419 (32.6%) | 84 (35.7%) | 2220 (32.7%) | 108 (37.0%) |
| 35-44 | 2072 (27.9%) | 71 (30.2%) | 1916 (28.2%) | 79 (27.1%) |
| 45-54 | 1248 (16.8%) | 41 (17.4%) | 1128 (16.6%) | 56 (19.2%) |
| 55+ | 906 (12.2%) | 21 (8.9%) | 791 (11.6%) | 25 (8.6%) |
| Gender |  |  |  |  |
| Male | 5460 (73.5%) | 234 (99.6%) | 4854 (71.4%) | 284 (97.3%) |
| Female | 1970 (26.5%) | 1 (0.4%) | 1942 (28.6%) | 8 (2.7%) |
| Nationality |  |  |  |  |
| Qatari | 1539 (20.7%) | 44 (18.7%) | 1419 (20.9%) | 63 (21.6%) |
| Non-Qatari | 5891 (79.3%) | 191 (81.3%) | 5377 (79.1%) | 229 (78.4%) |
| Marital status |  |  |  |  |
| Single | 1765 (24.0) | 47 (20.0%) | 1649 (24.3%) | 63 (21.6%) |
| Married | 5440 (73.9%) | 187 (79.6%) | 4991 (73.5%) | 228 (78.1%) |
| Divorced/widowed | 154 (2.1%) | 1 (0.4%) | 152 (2.2%) | 1 (0.3%) |
| Education |  |  |  |  |
| Secondary schooling or less | 3830 (52.3%) | 183 (78.2%) | 3439 (50.7%) | 204 (69.9%) |
| University | 3158 (43.1%) | 45 (19.2%) | 3033 (44.7%) | 78 (26.7%) |
| Postgraduate | 332 (4.5%) | 6 (2.6%) | 315 (4.6%) | 10 (3.4%) |
| Secondhand exposure |  |  |  |  |
| Smoke/smokeless inside home | 1040 (14.2%) | 73 (31.2%) | 925 (13.6%) | 42 (14.4%) |
| No | 6275 (85.8%) | 161 (68.8%) | 5863 (86.4%) | 250 (85.6%) |
|  |  |  |  |  |
| E-cigarettes inside home | 58 (0.8%) | 1 (0.4%) | 56 (0.8%) | 1 (0.3%) |
| No | 7257 (99.2%) | 233 (99.6%) | 6734 (99.2%) | 291 (99.7%) |
|  |  |  |  |  |
| Smoking/vaping at workplace | 543 (8.7%) | 27 (11.5%) | 486 (7.2%) | 30 (10.3%) |
| No | 5718 (78.2%) | 202 (86.3%) | 5271 (77.7%) | 243 (83.2%) |
| I do not work | 1052 (14.4%) | 5 (2.1%) | 1028 (15.2%) | 19 (6.5%) |
| Heart Diseases |  |  |  |  |
| Yes | 162 (2.2%) | 4 (1.7%) | 118 (1.7%) | 9 (3.1%) |
| No | 7264 (97.8%) | 231 (98.3%) | 6677 (98.3%) | 283 (96.9%) |
| Hypertension |  |  |  |  |
| Yes | 841 (11.3%) | 20 (8.5%) | 737 (10.8%) | 36 (12.3%) |
| No | 6585 (88.7%) | 215 (91.5%) | 6058 (89.2%) | 256 (87.7%) |
| Diabetes |  |  |  |  |
| Yes | 923 (12.4%) | 35 (14.9%) | 776 (11.4%) | 58 (19.9%) |
| No | 6503 (87.6%) | 200 (85.1%) | 6019 (88.6%) | 234 (80.1%) |
| Asthma |  |  |  |  |
| Yes | 244 (3.3%) | 6 (2.6%) | 228 (3.4%) | 8 (2.7%) |
| No | 7182 (96.7%) | 229 (97.4%) | 6567 (96.6%) | 284 (97.3%) |
| COPD |  |  |  |  |
| Yes | 5 (0.1%) | 0 (0.0%) | 2 (0.03%) | 0 (0.0%) |
| No | 7421 (9.9%) | 235 (100.0%) | 6793 (99.97%) | 292 (100.0%) |
| Cerebrovascular disease |  |  |  |  |
| Yes | 8 (0.1%) | 0 (0.0%) | 8 (0.1%) | 0 (0.0%) |
| No | 7418 (99.9%) | 235 (100.0%) | 6787 (99.9%) | 292 (100.0%) |
| Cancer |  |  |  |  |
| Yes | 16 (0.2%) | 0 (0.0%) | 14 (0.2%) | 2 (0.7%) |
| No | 7410 (99.8%) | 235 (100.0%) | 6781 (99.8%) | 290 (99.3%) |
| Obesity |  |  |  |  |
| Yes | 82 (1.1%) | 2 (0.9%) | 70 (1.0%) | 5 (1.7%) |
| No | 7344 (98.9%) | 233 (99.1%) | 6725 (99.0%) | 287 (98.3%) |
| Chronic renal disease |  |  |  |  |
| Yes | 86 (1.2%) | 1 (0.4%) | 41 (0.6%) | 0 (0.0%) |
| No | 7340 (98.8%) | 234 (99.6%) | 6754 (99.4%) | 292 (100.0%) |
| Others |  |  |  |  |
| Yes | 586 (7.9%) | 16 (6.8%) | 494 (7.3%) | 28 (9.6%) |
| No | 6840 (92.1%) | 219 (93.2%) | 6301 (92.7%) | 264 (90.4%) |
| Quarantine/Hospitalized |  |  |  |  |
| Quarantine | 5668 (76.3%) | 179 (76.2%) | 5284 (77.8%) | 203 (69.5%) |
| Hospitalized | 1762 (23.7%) | 56 (23.8%) | 1512 (22.2%) | 89 (30.5%) |
| Intensive Care Unit |  |  |  |  |
| Yes | 635 (8.5%) | 27 (11.5%) | 471 (6.9%) | 45 (15.4%) |
| No | 6795 (91.5%) | 208 (88.5%) | 6325 (93.1%) | 247 (84.6%) |
| Oxygen therapy |  |  |  |  |
| Yes | 839 (11.3%) | 31 (13.2%) | 655 (9.6%) | 54 (18.5%) |
| No | 6591 (88.7%) | 204 (86.8%) | 6141 (90.4%) | 238 (81.5%) |
| Mechanical ventilation |  |  |  |  |
| Yes | 328 (4.4%) | 20 (8.5%) | 212 (3.1%) | 17 (5.8%) |
| No | 7102 (95.6%) | 215 (91.5%) | 6584 (96.9%) | 275 (94.2%) |
| Outcome |  |  |  |  |
| Recovered without hospitalization | 5668 (76.3%) | 179 (76.2%) | 5284 (77.8%) | 203 (69.5%) |
| Recovered with hospitalization | 1636 (22.0%) | 52 (22.1%) | 1491 (21.9%) | 89 (30.5%) |
| Still in hospital | 7 (0.1%) | 1 (0.4%) | 6 (0.1%) | 0 (0.0%) |
| Death | 119 (1.6%) | 3 (1.3%) | 15 (0.2%) | 0 (0.0%) |

SD: Standard deviation *Includes daily and occasional users. ^Total does not add up due to missing data.

**S6 Table.** Sociodemographic and clinical characteristics of COVID-19 patients by electronic cigarette use status

|  | **COVID-19 patients** | | | |
| --- | --- | --- | --- | --- |
|  | All Patients^ | E-cigarette users* | E-cigarette non-users | E-cigarette ex-users |
|  | (n, % of total) | (n, %) | (n, %) | (n, %) |
| All subjects | 7430 (100%) | 42 (0.6%) | 7081 (96.9%) | 182 (2.5%) |
| Age (mean, ± SD) | 38.8 (±12.6) | 32.4 (±8.4) | 38.6 (±12.4) | 33.1 (8.5) |
| Age (years) |  |  |  |  |
| 18-24 | 785 (10.6%) | 4 (9.5%) | 753 (10.6%) | 25 (13.7%) |
| 25-34 | 2419 (32.6%) | 28 (66.7%) | 2283 (32.2%) | 94 (51.6%) |
| 35-44 | 2072 (27.9%) | 6 (14.3%) | 2012 (28.4%) | 43 (23.6%) |
| 45-54 | 1248 (16.8%) | 3 (7.1%) | 1203 (17.0%) | 17 (9.3%) |
| 55+ | 906 (12.2%) | 1 (2.4%) | 830 (11.7%) | 3 (1.6%) |
| Gender |  |  |  |  |
| Male | 5460 (73.5%) | 39 (92.9%) | 5155 (72.8%) | 171 (94.0%) |
| Female | 1970 (26.5%) | 3 (7.1%) | 1926 (27.2%) | 11 (6.0%) |
| Nationality |  |  |  |  |
| Qatari | 1539 (20.7%) | 10 (23.8%) | 1455 (20.5%) | 56 (30.8%) |
| Non-Qatari | 5891 (79.3%) | 32 (76.2%) | 5626 (79.5%) | 126 (69.2%) |
| Marital status |  |  |  |  |
| Single | 1765 (24.0) | 18 (42.9%) | 1656 (23.4%) | 79 (43.4%) |
| Married | 5440 (73.9%) | 23 (54.8%) | 5275 (74.5%) | 97 (53.3%) |
| Divorced/widowed | 154 (2.1%) | 1 (2.4%) | 146 (2.1%) | 6 (3.3%) |
| Education |  |  |  |  |
| Secondary schooling or less | 3830 (52.3%) | 10 (23.8%) | 3742 (52.9%) | 67 (36.8%) |
| University | 3158 (43.1%) | 28 (66.7%) | 3021 (42.7%) | 102 (56.0%) |
| Postgraduate | 332 (4.5%) | 4 (9.5%) | 314 (4.4%) | 13 (7.1%) |
| Secondhand exposure |  |  |  |  |
| Smoke/smokeless inside home | 1040 (14.2%) | 9 (21.4%) | 985 (13.9%) | 46 (25.3%) |
| No | 6275 (85.8%) | 33 (78.6%) | 6091(86.1%) | 136 (74.7%) |
|  |  |  |  |  |
| E-cigarettes inside home | 58 (0.8%) | 8 (19.0%) | 47 (0.7%) | 3 (1.6%) |
| No | 7257 (99.2%) | 34 (81.0%) | 7032 (99.3%) | 179 (98.4%) |
|  |  |  |  |  |
| Smoking/vaping at workplace | 543 (8.7%) | 8 (19.5%) | 508 (7.2%) | 26 (14.3%) |
| No | 5718 (78.2%) | 28 (68.3%) | 5531 (78.2%) | 146 (80.2%) |
| I do not work | 1052 (14.4%) | 5 (12.2%) | 1034 (14.6%) | 10 (5.5%) |
| Heart Diseases |  |  |  |  |
| Yes | 162 (2.2%) | 0 (0.0%) | 127 (1.8%) | 4 (2.2%) |
| No | 7264 (97.8%) | 42 (100.0%) | 6953 (98.2%) | 178 (97.8%) |
| Hypertension |  |  |  |  |
| Yes | 841 (11.3%) | 4 (9.5%) | 776 (11.0%) | 10 (5.5%) |
| No | 6585 (88.7%) | 38 (90.5%) | 6304 (89.0%) | 172 (94.5%) |
| Diabetes |  |  |  |  |
| Yes | 923 (12.4%) | 1 (2.4%) | 846 (11.9%) | 19 (10.4%) |
| No | 6503 (87.6%) | 41 (97.6%) | 6234 (88.1%) | 163 (89.6%) |
| Asthma |  |  |  |  |
| Yes | 244 (3.3%) | 2 (4.8%) | 227 (3.2%) | 12 (6.6%) |
| No | 7182 (96.7%) | 40 (95.2%) | 6853 (96.8%) | 170 (93.4%) |
| COPD |  |  |  |  |
| Yes | 5 (0.1%) | 0 (0.0%) | 2 (0.03%) | 0 (0.0%) |
| No | 7421 (9.9%) | 42 (100.0%) | 7078 (99.97%) | 182 (100.0%) |
| Cerebrovascular disease |  |  |  |  |
| Yes | 8 (0.1%) | 0 (0.0%) | 8 (0.1%) | 0 (0.0%) |
| No | 7418 (99.9%) | 42 (100.0%) | 7072 (99.9%) | 182 (100.0%) |
| Cancer |  |  |  |  |
| Yes | 16 (0.2%) | 0 (0.0%) | 15 (0.2%) | 1 (0.5%) |
| No | 7410 (99.8%) | 42 (100.0%) | 7065 (99.8%) | 181 (99.5%) |
| Obesity |  |  |  |  |
| Yes | 82 (1.1%) | 0 (0.0%) | 75 (1.1%) | 2 (1.1%) |
| No | 7344 (98.9%) | 42 (100.0%) | 7005 (98.9%) | 180 (98.9%) |
| Chronic renal disease |  |  |  |  |
| Yes | 86 (1.2%) | 0 (0.0%) | 41 (0.6%) | 0 (0.0%) |
| No | 7340 (98.8%) | 42 (100.0%) | 7039 (99.4%) | 182 (100.0%) |
| Others |  |  |  |  |
| Yes | 586 (7.9%) | 2 (4.8%) | 522 (7.4%) | 12 (6.6%) |
| No | 6840 (92.1%) | 40 (95.2%) | 6558 (92.6%) | 170 (93.4%) |
| Quarantine/Hospitalized |  |  |  |  |
| Quarantine | 5668 (76.3%) | 34 (81.0%) | 5470 (77.2%) | 149 (81.9%) |
| Hospitalized | 1762 (23.7%) | 8 (19.0%) | 1611 (22.8%) | 33 (18.1%) |
| Intensive Care Unit |  |  |  |  |
| Yes | 635 (8.5%) | 0 (0.0%) | 535 (7.6%) | 5 (2.7%) |
| No | 6795 (91.5%) | 42 (100.0%) | 6546 (92.4%) | 177 (97.3%) |
| Oxygen therapy |  |  |  |  |
| Yes | 839 (11.3%) | 1 (2.4%) | 727 (10.3%) | 7 (3.8%) |
| No | 6591 (88.7%) | 41 (97.6%) | 6354 (89.7%) | 175 (96.2%) |
| Mechanical ventilation |  |  |  |  |
| Yes | 328 (4.4%) | 0 (0.0%) | 243 (3.4%) | 1 (0.5%) |
| No | 7102 (95.6%) | 42 (100.0%) | 6838 (96.6%) | 181 (99.5%) |
| Outcome |  |  |  |  |
| Recovered without hospitalization | 5668 (76.3%) | 34 (81.0%) | 5470 (77.2%) | 149 (81.9%) |
| Recovered with hospitalization | 1636 (22.0%) | 8 (19.0%) | 1589 (22.4%) | 33 (18.1%) |
| Still in hospital | 7 (0.1%) | 0 (0.0%) | 7 (0.1%) | 0 (0.0%) |
| Death | 119 (1.6%) | 0 (0.0%) | 15 (0.2%) | 0 (0.0%) |

SD: Standard deviation *Includes daily and occasional users. ^Total does not add up due to missing data.

**S7 Table.** The number and percentage of different types of comorbidities (n, %)

|  | **N=7430** | **Prevalence (95% CI)** |
| --- | --- | --- |
| **Comorbidities** |  |  |
| None | 5403 | 72.7 (71.7-73.7) |
| Any | 2027 | 27.3 (26.3-28.3) |
| **Number of comorbidities** |  |  |
| 1 | 1372 | 18.5 (17.6-19.3) |
| 2 | 444 | 6.0 (5.4-6.5) |
| 3 or more | 211 | 2.8 (2.5-3.2) |
| **Most common comorbidities*** |  |  |
| Heart Diseases | 162 | 2.2 (1.8-2.5) |
| Hypertension | 841 | 11.3 (10.6-12.0) |
| Diabetes | 923 | 12.4 (11.7-13.2) |
| Asthma | 244 | 3.3 (2.9-3.7) |
| Chronic Obstructive Pulmonary Disease | 5 | 0.07 (0.008-0.13) |
| Cardiovascular Disease | 8 | 0.10 (0.03-0.18) |
| Cancer | 16 | 0.22 (0.11-0.32) |
| Chronic renal disease | 86 | 1.16 (0.9-1.4) |
| Obesity | 82 | 1.10 (0.9-1.4) |
| Others | 586 | 7.9 (7.3-8.5) |

*The same subject may be listed in more than 1 category

**S8 Table.** Hospitalized smokeless tobacco users among mild and severe COVID-19 patients by nationality (n, %)

| Nationality | Mild (N=27) | % | Severe (N=29) | % |
| --- | --- | --- | --- | --- |
| Qataris | 6 | 22.22 | 1 | 3.45 |
| Afghan | 2 | 7.41 | 0 | - |
| Bangladesh | 0 | - | 4 | 13.79 |
| Indian | 7 | 25.93 | 3 | 10.34 |
| Nepali | 4 | 14.81 | 16 | 55.17 |
| Omani | 1 | 3.70 | 0 | - |
| Pakistani | 4 | 14.81 | 4 | 13.79 |
| Sri Lankan | 1 | 3.70 | 0 | - |
| Sudanese | 2 | 7.41 | 1 | 3.45 |

**S9 Table.** Hospitalized smokeless tobacco users by severity of COVID-19 outcome (n, %)

| Outcome of the case | Mild (N=27) | % | Severe (N=29) | % |
| --- | --- | --- | --- | --- |
| Discharged from hospital and recovered | 27 | 100% | 25 | 86.21 |
| Still in hospital | 0 | 0 | 1 | 3.45 |
| Death | 0 | 0 | 3 | 10.34 |

**S10 Table.** Smokeless tobacco users with severe COVID-19 by hospitalization characteristics

| Severe COVID19 patients by smokeless use |  | N | Mean | Standard deviation | F | p-value |
| --- | --- | --- | --- | --- | --- | --- |
| Non-user | Hospital (days) | 473 | 27.52 | 25.96 | 0.187 | 0.830 |
|  | ICU (days) | 453 | 10.36 | 10.62 |  |  |
|  | Mechanical ventilation (days) | 192 | 8.92 | 9.99 |  |  |
|  |  |  |  |  |  |  |
| User | Hospital (days) | 28 | 25.82 | 18.04 | 0.216 | 0.806 |
|  | ICU (days) | 27 | 11.67 | 16.29 |  |  |
|  | Mechanical ventilation (days) | 18 | 8.72 | 10.62 |  |  |
|  |  |  |  |  |  |  |
| Ex-user | Hospital (days) | 44 | 29.43 | 21.40 | 0.480 | 0.619 |
|  | ICU (days) | 42 | 9.98 | 10.05 |  |  |
|  | Mechanical ventilation (days) | 16 | 6.44 | 3.69 |  |  |

* Factors with p-value < 0.05 were considered statistically significant.

**S11 Table.** Multivariable logistic regression of hospitalized and severe COVID-19 patients among less than 50 years old

| **Characteristic** | **Hospitalized**  **Adjusted Odds Ratio (AOR) (95% CI)** | **p-value*** | **Severe Covid-19**  **Adjusted Odds Ratio (AOR) (95% CI)** | **p-value*** |
| --- | --- | --- | --- | --- |
| Gender |  |  |  |  |
| Female | 1.00 (Ref.) | - | 1.00 (Ref.) | - |
| Male | 1.1 (1.0-1.4) | 0.123 | 2.7 (1.8-4.1) | <0.001 |
| Nationality |  |  |  |  |
| Qatari | 1.00 (Ref.) | - | 1.00 (Ref.) | - |
| Non-Qatari | 1.4 (1.2-1.7) | <0.001 | 2.4 (1.5-4.1) | <0.001 |
| Tobacco Smoking |  |  |  |  |
| Never-smoker | 1.00 (Ref.) | - | 1.00 (Ref.) | - |
| Smoker | 0.6 (0.4-0.7) | <0.001 | 0.2 (0.1-0.4) | <0.001 |
| Ex-smoker | 1.2 (1.0-1.4) | 0.042 | 0.7 (0.5-1.0) | 0.054 |
| Smokeless (sweika/paan) |  |  |  |  |
| Never-user | 1.00 (Ref.) | - | 1.00 (Ref.) | - |
| User | 1.2 (0.8-1.7) | 0.421 | 2.7 (1.3-5.5) | 0.006 |
| Ex-user | 1.5 (1.1-2.1) | 0.011 | 2.4 (1.4-4.1) | 0.002 |
| Heart Diseases |  |  |  |  |
| No | 1.00 (Ref.) | - | 1.00 (Ref.) | - |
| Yes | 3.1 (1.7-5.9) | <0.001 | 5.2 (1.9-14.6) | 0.002 |
| Hypertension |  |  |  |  |
| No | 1.00 (Ref.) | - | 1.00 (Ref.) | - |
| Yes | 2.2 (1.8-2.9) | <0.001 | 1.7 (1.1-2.5) | 0.013 |
| Diabetes |  |  |  |  |
| No | 1.00 (Ref.) | - | 1.00 (Ref.) | - |
| Yes | 3.5 (2.8-4.3) | <0.001 | 2.3 (1.6-3.3) | <0.001 |
| Asthma |  |  |  |  |
| No | 1.00 (Ref.) | - | - | - |
| Yes | 1.7 (1.2-2.4) | 0.005 | - | - |
| Obesity |  |  |  |  |
| No | 1.00 (Ref.) | - | - | - |
| Yes | 1.5 (0.8-2.8) | 0.263 | - | - |
| Chronic renal disease |  |  |  |  |
| No | 1.00 (Ref.) | - | 1.00 (Ref.) | - |
| Yes | 6.0 (2.1-16.9) | <0.001 | 1.7 (0.4-7.1) | 0.443 |
| Other diseases |  |  |  |  |
| No | 1.00 (Ref.) | - | 1.00 (Ref.) | - |
| Yes | 2.1 (1.6-2.6) | <0.001 | 1.9 (1.2-2.9) | 0.005 |

* Factors with p-value < 0.05 were considered statistically significant.

**S12 Table.** Multivariable logistic regression of hospitalized and severe COVID-19 patients among 50 years and above

| **Characteristic** | **Hospitalized**  **Adjusted Odds Ratio (AOR) (95% CI)** | **p-value*** | **Severe Covid-19**  **Adjusted Odds Ratio (AOR) (95% CI)** | **p-value*** |
| --- | --- | --- | --- | --- |
| Gender |  |  |  |  |
| Female | 1.00 (Ref.) | - | 1.00 (Ref.) | - |
| Male | 1.5 (1.2-2.6) | 0.002 | 2.0 (1.3-3.0) | 0.002 |
| Nationality |  |  |  |  |
| Qatari | - | - | 1.00 (Ref.) | - |
| Non-Qatari | - | - | 3.2 (2.1-5.0) | <0.001 |
| Tobacco Smoking |  |  |  |  |
| Never-smoker | 1.00 (Ref.) | - | - | - |
| Smoker | 0.3 (0.2-0.5) | <0.001 | - | - |
| Ex-smoker | 1.0 (0.8-1.3) | 0.960 | - | - |
| Heart Diseases |  |  |  |  |
| No | 1.00 (Ref.) | - | 1.00 (Ref.) | - |
| Yes | 2.2 (1.3-3.5) | 0.001 | 1.9 (1.1-3.2) | 0.018 |
| Hypertension |  |  |  |  |
| No | 1.00 (Ref.) | - | - | - |
| Yes | 1.7 (1.4-2.2) | <0.001 | - | - |
| Diabetes |  |  |  |  |
| No | 1.00 (Ref.) | - | 1.00 (Ref.) | - |
| Yes | 2.5 (1.9-3.2) | <0.001 | 1.5 (1.1-2.1) | 0.017 |
| Cancer |  |  |  |  |
| No | 1.00 (Ref.) | - | - | - |
| Yes | 7.0 (1.4-33.9) | 0.016 | - | - |
| Chronic renal disease |  |  |  |  |
| No | 1.00 (Ref.) | - | 1.00 (Ref.) | - |
| Yes | 6.9 (2.8-16.9) | <0.001 | 6.4 (3.0-13.8) | <0.001 |
| Other diseases |  |  |  |  |
| No | 1.00 (Ref.) | - | 1.00 (Ref.) | - |
| Yes | 2.0 (1.4-2.8) | <0.001 | 2.8 (1.8-4.3) | <0.001 |

* Factors with p-value < 0.05 were considered statistically significant.
